# Stream Vulnerability to Widespread and Emergent Stressors: A Focus on Unconventional Oil and Gas

**DOI:** 10.1371/journal.pone.0137416

**Published:** 2015-09-23

**Authors:** Sally A. Entrekin, Kelly O. Maloney, Katherine E. Kapo, Annika W. Walters, Michelle A. Evans-White, Kenneth M. Klemow

**Affiliations:** 1 Biology Department, University of Central Arkansas, Conway, Arkansas, United States of America; 2 Northern Appalachian Research Laboratory, U. S. Geological Survey Leetown Science Center, Wellsboro, Pennsylvania, United States of America; 3 Waterborne Environmental Inc., Leesburg, Virginia, United States of America; 4 U.S. Geological Survey, Wyoming Cooperative Fish and Wildlife Research Unit, Department of Zoology and Physiology, University of Wyoming, Laramie, Wyoming, United States of America; 5 Department of Biological Sciences, University of Arkansas, Fayetteville, Arkansas, United States of America; 6 Department of Biology, Wilkes University, Wilkes-Barre, Pennsylvania, United States of America; Northwest Fisheries Science Center, UNITED STATES

## Abstract

Multiple stressors threaten stream physical and biological quality, including elevated nutrients and other contaminants, riparian and in-stream habitat degradation and altered natural flow regime. Unconventional oil and gas (UOG) development is one emerging stressor that spans the U.S. UOG development could alter stream sedimentation, riparian extent and composition, in-stream flow, and water quality. We developed indices to describe the watershed sensitivity and exposure to natural and anthropogenic disturbances and computed a vulnerability index from these two scores across stream catchments in six productive shale plays. We predicted that catchment vulnerability scores would vary across plays due to climatic, geologic and anthropogenic differences. Across-shale averages supported this prediction revealing differences in catchment sensitivity, exposure, and vulnerability scores that resulted from different natural and anthropogenic environmental conditions. For example, semi-arid Western shale play catchments (Mowry, Hilliard, and Bakken) tended to be more sensitive to stressors due to low annual average precipitation and extensive grassland. Catchments in the Barnett and Marcellus-Utica were naturally sensitive from more erosive soils and steeper catchment slopes, but these catchments also experienced areas with greater UOG densities and urbanization. Our analysis suggested Fayetteville and Barnett catchments were vulnerable due to existing anthropogenic exposure. However, all shale plays had catchments that spanned a wide vulnerability gradient. Our results identify vulnerable catchments that can help prioritize stream protection and monitoring efforts. Resource managers can also use these findings to guide local development activities to help reduce possible environmental effects.

## Introduction

Healthy streams support drinking water, recreation, and fisheries, yet, 79% of assessed U.S. river and stream miles have degraded environmental conditions with significantly altered biological communities [1]. Excess phosphorus and nitrogen, sediment, metals, and other contaminants from agriculture, urbanization, and wastewater are common stream stressors that degrade water and biological quality [1]. Degradation can also result from hydrologic changes, including reduced in-stream flows from water withdrawals for agriculture, recreation, human consumption, and energy development or increased stream flashiness as impervious surfaces increase in the surrounding catchment. Energy development, for example coal mining, oil and gas extraction, and nuclear energy, have altered stream quality through water withdrawals, channel modification such as channelizing or filling, altered flow paths and runoff and contaminants such as heavy metals and sediment [2–6]. A rapidly expanding source of energy such as unconventional oil and gas extraction (UOG) from shale has the potential to alter streams through water withdrawals, land development, spills and wastewater production. Currently, it is not known how and to what extent this emerging land use will interact with existing stressors to influence the quality and function of stream ecosystems [7].

Global demand for energy, together with recent technological advances, has led to increased development of oil and gas from shale and coalbed methane sources [8,9]. Shale is a large reservoir of oil and gas with technically recoverable estimates from 41 countries at 7,299 trillion cubic feet (TCF) of natural gas and 345 billion barrels of oil [10]. The U.S. leads the way in developing this resource with a 6000% (~2–11.9 TCF) increase in U.S. shale natural gas production from 2007 to 2013 [11]. Coalbed methane is another large source of natural gas in the U.S., with 2013 production levels at 1.4 TCF. In 2013, the U.S. extracted 30.0 TCF of natural gas with 39.6% from shale and 4.7% from coalbed reservoirs (U.S. DOE/EIA 2014). The U.S. is estimated to only have 9.1% (665 TCF) of the world’s technically recoverable shale gas and 16.8% (58 BBL) of the world’s technically recoverable shale oil [10]. China (15.3% natural gas; 9.3% oil), Argentina (11.0% natural gas; 7.8% oil), Algeria (9.7% natural gas), Russia (3.9% gas; 21.7% oil), and other European countries (http://www.eia.gov/analysis/studies/worldshalegas/pdf/fullreport.pdf) have significant untapped reserves that will likely be developed in the future. In the contiguous U.S., the Northern Appalachian plays have the highest estimated total recoverable shale gas reserves (Table 1) followed by the Hilliard-Baxter-Mancos play, and then the Fayetteville and Barnett plays; the Niobrara-Mowry play has the lowest estimated shale gas reserves and is currently producing mostly coalbed methane (http://wogcc.state.wy.us/ ). The Bakken play has the largest estimated total recoverable reserves for shale oil in the contiguous U.S. (7.4 billion barrels). A broad framework that aims to reduce stream exposure to these emerging stressors and minimize interactions with current and widespread land uses would benefit resource management [12].

**Table 1:** Estimated size and recoverable unconventional oil or gas in each shale play. TCF—trillion cubic feet.

Shale Basin (from EIA)	Shale Play (from EIA)	Area (km2) from EIA shapefile	Shale gas (TCF)	Natural Gas liquids (billion barrels)	Shale oil (billion barrels)	Year Assessed
Appalachian	Marcellus^1^	201,168	84.2	3.4	——	2011
Appalachian	Devonian^2^	141,494	——	——	——	
Appalachian	Utica^3^	232,404	38.2	0.21	0.94	2012
	Total	575,066				
Arkoma	Fayetteville^4^	15,158	38	0.16	0	2010
Ft. Worth	Barnett^5^	68,146	26.7	1.1	0.1	2003
Williston	Bakken^6^	96,791	6.7	0.53	7.4	2013
Powder River	Niobrara-Mowry ^7^	22,691	2.5	0.05	0	2002
Greater Green River	Hilliard Baxter Mancos-Niobrara^8^	46,445	84.6	2.6	0.13	2002

^1^ http://www.usgs.gov/newsroom/article.asp?ID=2893.

^2^ http://pubs.usgs.gov/fs/2011/3092/pdf/fs2011-3092.pdfhttp://pubs.usgs.gov/fs/fs-009-03/.

^3^ http://www.usgs.gov/newsroom/article.asp?ID=3419&from=rss_home.

^4^ http://pubs.usgs.gov/fs/2010/3043/.

^5^ http://pubs.usgs.gov/fs/2004/3022/.

^6^ http://www.doi.gov/news/pressreleases/usgs-releases-new-oil-and-gas-assessment-for-bakken-and-three-forks.

^7^ http://energy.usgs.gov/OilGas/AssessmentsData/NationalOilGasAssessment/USBasinSummaries.aspx?provco.

^8^ http://pubs.usgs.gov/fs/fs-145-02/fs-145-02.html.

The spatial extent and fast pace of unconventional oil and natural gas development (UOG) has raised concerns about its ecological effects on both terrestrial and aquatic ecosystems [13–18]. Disturbances to aquatic communities could occur from infrastructure development, water use, or environmental contamination associated with fracturing, waste collection, and/or disposal [19–21]. Infrastructure development may cause fine sedimentation from construction of well pads, roads, and pipelines and from reduced riparian vegetation and associated habitat fragmentation [15]. Water resources are already over-allocated in many regions and excessive water withdrawals for UOG could place additional pressure on maintenance of flows that sustain natural biological communities and processes [22–24]. Environmental contamination can be accidental from leaking wastewater storage systems, fluid-truck or train accidents, pipeline ruptures, inadequate water treatment or spreading brine on fields and roads [18, 25–28]. UOG development has been rapid, and no peer-reviewed studies have examined the probability or occurrence of cumulative ecological effects on aquatic ecosystems across shale plays [7].

There is a small but growing literature showing the relationship between UOG development and the ecology of aquatic ecosystems [28–30]. Ecological effects may not be broadly applicable across shale plays because ecoregions are characterized by different environmental conditions and human activities. Slope, geology, soil type, and vegetation can all vary across biome types and can affect water, sediment, and contaminant movement from areas experiencing UOG development[31–33]. Climate, such as the amount of precipitation and seasonality, can affect the catchment sensitivity to water use and the transfer of contaminants associated with UOG activities. Ecosystem vulnerability to UOG development will also likely depend on the extent and interaction among other existing landscape stressors (e.g., agriculture, urbanization, and mining). Current measures of vulnerability can be used to predict future ecosystem degradation with the addition of emerging stressors. Current U.S. UOG plays span an environmental gradient from semi-arid Western plays with lower gradient catchments and less precipitation to Eastern plays (Barnett, Fayetteville, and Marcellus) with greater annual precipitation and more contiguous forested vegetation. The natural environmental conditions and existing stressors will likely influence ecological effects on aquatic communities from UOG activities [23].

In addition to variation in background environmental conditions and existing exposure to anthropogenic landscape stressors, the extent and intensity of energy development varies among existing plays [34]. Five shale plays in the U.S. are currently supplying 80% of all shale gas in the United States. The Appalachian basin (Marcellus and Utica plays) is currently producing the most natural gas, and the Williston basin (Bakken play) is currently producing the most oil [10]. Unconventional oil and gas development has expanded rapidly in some Northern Appalachian states, like Pennsylvania and West Virginia, while New York has recently banned UOG development and the Delaware River Basin of eastern Pennsylvania has placed a moratorium on UOG extraction pending more research [35]. Environmental context within plays is expected to interact with the intensity and extent of UOG development to affect the efficacy of best management practices such as setbacks and erosion control.

Vulnerability metric or threat index approaches have been used to address many management issues including agriculture, urbanization, and mining [36]. Vulnerability can be characterized as a combination of the **exposure** of the ecosystem to anthropogenic *stressors*, such as well pads, impervious surfaces, roads, mines, and agriculture and the natural **sensitivity** of that ecosystem to alterations. Ecosystems with the greatest natural sensitivity and exposure to stressors should be most vulnerable to current and added stressors [36]. Organisms within vulnerable ecosystems have the greatest risk of extinction where sensitive taxa could already be lost or they are more likely to be lost with added stressors [37]. Bioassessments are traditionally conducted in selected ecosystems to quantify biological degradation from anthropogenic activities. Ecosystem sensitivity and vulnerability models can flag at-risk ecosystems to prioritize the needed biological sampling for such bioassessments. An UOG vulnerability index could simultaneously provide both a starting point to examine UOG-related effects on freshwater biota and a cross-state metric to guide best management practices for UOG development.

The location of UOG development coupled with a suite of environmental conditions provide a testable framework to identify surface waters more or less vulnerable to current and future stressors. These “vulnerable” waters could be identified by development of a multivariate threat index. A comparison among metrics that make plays more or less sensitive to activities associated with UOG will help predict the potential ecological effects within a catchment. For example, catchments with high density and extent of unconsolidated sediment are more likely to transport contaminants from source to nearby streams. Additionally, catchments receiving more rainfall could be less vulnerable to low-flows if water is withdrawn for hydraulic fracturing.

Our first objective was to characterize the amount of UOG development across plays within the context of other natural and anthropogenic variables and then develop a vulnerability index composed of sensitivity and exposure metrics for six productive shale plays across the contiguous U.S. Variables incorporated into the index were chosen based on their known effect on streams and their ubiquity across the landscape, relationship to gas activity, and data accessibility at a hydrologic unit code 12 (HUC12) resolution [36]. Variable incorporation was informed by peer reviewed literature, and agreed upon by author experience in each shale basin and informed by other freshwater studies [36,38]. Our second objective was to compute a play-average HUC12 vulnerability to three main postulated UOG alterations: natural flow regime, sedimentation, and chemical stressors [17,39]. We used the play average sensitivity and severity-weighted exposure scores to estimate vulnerability based on the methods of [7, 36] for each play.

## Methods

### Study site

**Fig 1:**
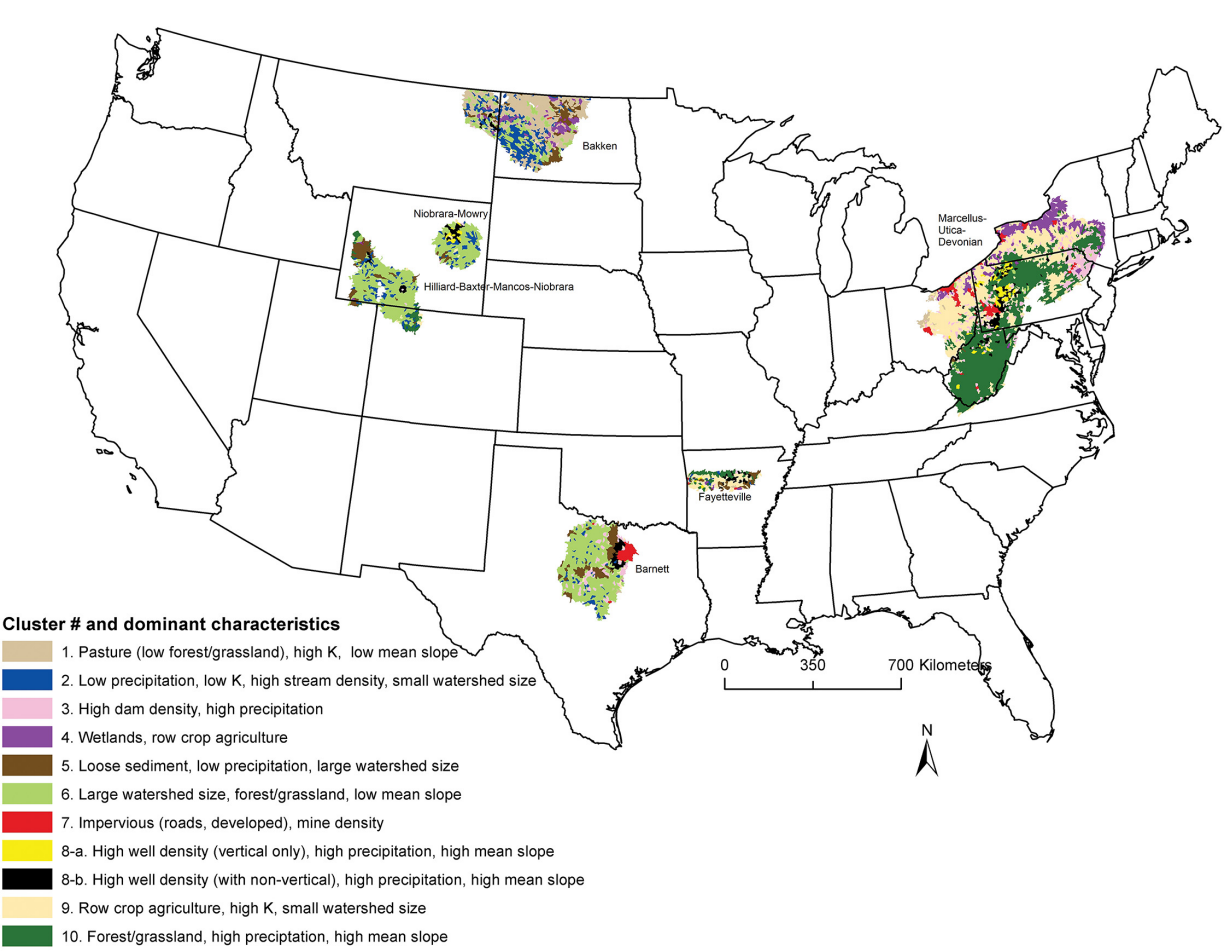
Land cover and land use values are shown as cluster results for HUC12s. Cluster scores were mapped to visualize geographic trends and descriptive statistics were computed to identify unique characteristics of the ten cluster groups. Refer to S2 Table for cluster results.

We selected six major shale play areas across the contiguous U.S. (Fig 1) to assess the vulnerability of aquatic ecosystems to shale gas development. The major shale plays were the Marcellus, Utica and Upper Devonian plays of the mid-Atlantic region (Appalachian Basin), the Fayetteville shale primarily located in Arkansas (Arkoma Basin), the U.S. portion of the Bakken shale in North Dakota and Montana (Williston Basin), the Barnett shale located in Texas (Ft. Worth Basin), the Niobrara-Mowry shale play in Wyoming (Powder River Basin), and the Hilliard-Baxter-Mancos shale play of Wyoming and Colorado (Greater Green River Basin). The Northern Appalachian plays primarily span the Appalachian mixed mesophytic forests and the Allegheny highlands forest designated as ecoregion 8 (Eastern Temperate Forests, Level I, CEC, 1997). The Fayetteville play primarily underlies Ozark mountain forest within the Eastern Temperate forest. The Bakken play underlies the northern short and mixed grasslands. The Barnett play underlies mostly the Central forest-grasslands transition and expands over to the Central and Southern mixed grasslands. Bakken and Barnett plays are ecoregion 9 (Great Plains). The Niobrara-Mowry play primarily spans Northern short grasslands, while the Hilliard-Baxter-Mancos is mainly short grass and shrub steppe. These two Western plays span the Great Plains (Level 9), North American Desert (Level 10), and Northwestern Forested Mountains (Level 6). Our level of analysis was at the HUC12 scale; catchments of that scale encompass 1–792 km^2^, averaging 92 km^2^. We selected each HUC12 that intersected the oil and gas play of interest. For each HUC12, we calculated a set of environmental characteristics or exposures that could affect aquatic ecosystems and eliminated highly correlated variables (Spearman rho r>0.6).

### Measures of the extent of UOG development, well densities and proximity to surface waters

We collected well point data from various State, non-profit, and for-profit on-line databases, and we only included wells that were classified as active, abandoned or in drilling stage (See S1 Table for detailed State-by-State well evaluation and selection procedures). As a measure of shale oil and gas development in a given HUC12, we calculated the number and density (number m^-2^) of all gas, oil, or oil and gas wells, vertical only wells, and non-vertical (horizontal and directional) wells that have occurred in each shale play area from 01 January 2000 through 31 December 2012. We used non-vertical wells as a proxy for UOG development because not all States reported unconventional or not. Vertical wells also may be fractured; however, they usually only constitute a small proportion to the overall UOG footprint. For example, in Pennsylvania from 01 January 2010 through 31 December 2014, 13,271 wells were spudded. 6,066 wells were listed as vertical of which only 426 were listed as unconventional. 7,078 wells were listed as horizontal of which 7,062 were unconventional. We quantified the area that currently has UOG (i.e. non-vertical wells) and conventional natural gas (CNG) wells for each play using the extent of coverage tool in ArcMap (ESRI, Redlands, California, USA). Minimum, maximum, and average proximity of all surface well locations in each HUC12 to nearest NHDPlusV2 surface waters were generated in ArcMap with the near tool in the proximity analysis tool set. HUC12s without any wells were only omitted in the proximity analyses, but all HUC12s were included in all other analyses. Data for other infrastructure associated with shale oil and gas development (e.g., well pads, pipelines and access roads) were not uniformly or consistently available across our study area. However, these variables are often positively related to well density, which was identified as a reasonable indicator of overall shale oil and gas development in Fayetteville Shale catchments [28]. Thus, well density represented the extent and intensity of all gas activity within a catchment.

### Sensitivity, exposure, and vulnerability calculations

Parameters that will reflect ecosystem natural sensitivity to all UOG infrastructure (well pads, roads, and pipelines) included thirty year mean precipitation (mm), catchment slope (degrees), % wetlands, % vegetation cover (forest + grassland), % unconsolidated sediment, soil K factor (indicates erodibility), and stream density (km km^-2^) in each HUC12 [36]. HUC12 sensitivity ranged from 0–4, with 4 as the most sensitive. Sensitivity scores were assigned based on calculated quartiles using cumulative distribution from all HUC12s, where a mean of zero was ranked as 0, 25^th^ percentile as 1, 50^th^ percentile as 2 and so on, with the exception of precipitation. For precipitation, the scoring was inversed so that lower precipitation was ranked as more sensitive. In the absence of scientific studies to guide scoring, percentile scoring is a reasonable first step [36]. Total sensitivity was then calculated for each HUC12 as the sum of each parameter’s sensitivity score.

Exposure variables were chosen based on the availability of a national coverage and potential to alter surface water quality [40–42]. Anthropogenic exposure variables were paved road density (km km^-2^), % impervious surface cover, % crop cover, % pasture cover, mining of metallic and nonmetallic resources (hereafter, mine density (# km^-2^), dam density (# km km^-2^), vertical well density (# km^-2^), average vertical and non-vertical well proximity to NHD flowlines, and non-vertical well density (# km^-2^). Watershed exposure was scored in a similar manner to the sensitivity metrics (0–4, based on quartiles of values) with the exception of agriculture and impervious surface cover whose rankings were taken from published literature [36]. Well proximity to NHDPlus flowlines were scored with lower values corresponding to a higher exposure. Zeros were included as a potential score for all variables except proximity of wells to flowlines. Total exposure was then calculated for each HUC12 as the sum of each parameter’s exposure score. Data sources for all variables used to rank sensitivity and exposure are summarized in Table 2. Vulnerability for each HUC12 was estimated by multiplying total sensitivity and exposure [35].

**Table 2:** Variables included in cluster analysis and vulnerability index calculations as indicated by an * and data sources. NLCD–National Land Cover Database.

Variable Type	Variable Description	Data Source
General watershed characteristics	Catchment area (km^2^)	HUC12 area from the NHD; http://datagateway.nrcs.usda.gov/
	Average elevation in each HUC12 (m)	100 m digital elevation model; http://nationalatlas.gov/atlasftp.html#elev48i
Characteristics that increase watershed sensitivity	Average slope in each HUC12 (percent)*	Slope raster calculated from the 100 m DEM in ArcGIS.
	Precipitation (mm)*	PRISM 30 year normals; http://www.prism.oregonstate.edu/normals/
	Soil erodibility factor *	STATSCO soils data for the Conterminous United States; http://water.usgs.gov/GIS/metadata/usgswrd/XML/ussoils.xml
	Drainage density of NHDplus flowlines in each HUC12 watershed (km/km^2^)*	NHDplus data; http://www.horizon-systems.com/nhdplus/
	% Wetlands * (NLCD class 90 + 95)	2006 NLCD datasets; http://www.mrlc.gov/nlcd06_data.php
	% Forest + %Grassland* (NLCD class 41 + 42 + 43)	2006 NLCD datasets; http://www.mrlc.gov/nlcd06_data.php
	Proportion of loose sediment*	http://tin.er.usgs.gov/geology/state/ Sum of LITH6 categories “sand”, “gravel”, and “dune sand”, “alluvium”, “alluvial terrace”, “glacial drift”, “unconsolidated sediments”
Potential stressors	Well density (all, wells/km^2^)	Well data came from each states oil and gas website; we only selected wells that were labeled as "oil or gas well", " that were drilled post 1 January 2000, that were "active" or "Abandoned." See Appendix 1 for a detailed description of well data sources and selection procedures.
	Well density (vertical, wells/km^2^)*	
	Well density (non-vertical, wells/km^2^)*	
	Well count (all)	
	Well count (vertical)	
	Well count (non-vertical)	
	Dam Density (#/km^2^)*	NID database: http://geo.usace.army.mil/pgis/f?p=397:12
	Mine Density (#/km^2^)*	USGS Mineral Resources Database: http://tin.er.usgs.gov/mrds/
	Road density (km/km^2^)*	TIGER 2010 Streets; http://datagateway.nrcs.usda.gov/GDGOrder.aspx?order=QuickState
	% Impervious surface*	2006 NLCD datasets; http://www.mrlc.gov/nlcd06_data.php
	% Developed (NLCD class 21 + 22 + 23 + 24)	2006 NLCD datasets; http://www.mrlc.gov/nlcd06_data.php
	% High-intensity Developed (NLCD class 24)	2006 NLCD datasets; http://www.mrlc.gov/nlcd06_data.php
	% Pasture * (NLCD class 81)	2006 NLCD datasets; http://www.mrlc.gov/nlcd06_data.php
	% Cultivated crops* (NLCD class 82)	2006 NLCD datasets; http://www.mrlc.gov/nlcd06_data.php

The above sensitivity, exposure and vulnerability analysis provides a useful framework to rank individual HUC12s and average shale play vulnerability to UOG development; however, it does not directly rank each play’s average vulnerability to the three main postulated effects of UOG on streams: altered natural flow regime, sedimentation, and chemical contamination [17,42]. To make these more direct connections, we developed an average shale play severity-weighted index following the methods of [7, 36]. Briefly, authors ranked each exposure variable from 1 (lowest potential to cause stress) to 3 (highest potential to cause stress) for each of the three postulated effects of UOG. Scores were then averaged and used to weight exposure scores. For example, the non-vertical well density author-averaged natural flow regime severity score was 2.00. Exposure scores from the vulnerability analysis for non-vertical wells ranked the plays Mowry<Marcellus<Hilliard<Bakken<Barnett<Fayetteville; the numerical rankings were then multiplied by 2.00 for the weighted exposure score. So for non-vertical wells the weighted exposure score for the susceptibility analysis was 2 for Mowry, 4 for Marcellus, 6 for Hilliard, 8 for Bakken, 10 for Barnett and 12 for Fayetteville. We also only included those variables that were most widespread across the shale plays of interest. Ranked sensitivity variables were summed and severity-weighted exposure scores were summed and then the two scores were multiplied to derive a total severity-weighted index. For example, the Bakken had a total sensitivity score of 9 (4 for precipitation and 5 for stream density) and a total weighted exposure score of 51. Severity-weighted vulnerability to a change in the natural flow regime from UOG in the Bakken was 459. While our approach is simplistic and ignores some important variables, it highlights patterns that could support future and needed research.

### Data analysis

#### Cluster analysis

An exploratory cluster analysis was performed to better visualize trends in “similar” environmental conditions across plays (i.e., combinations of the exposure/sensitivity variables per HUC12 detailed in Table 2). A robust variation of the K-means clustering technique (K- medoids, “pam” function in R package ‘cluster’, [43]) was applied to the data based on 10 clusters (number determined by optimal average silhouette width, “pamk” function in R package ‘fpc’, [44]). Cluster results for HUC12s were mapped to visualize geographic trends. Descriptive statistics were computed to identify unique characteristics of the ten cluster groups.

#### Analysis of variables driving differences in sensitivity and exposure

The weight that any one variable had on sensitivity or exposure values was calculated as the percentage of change from the original score (i.e. original score-modified score/original score x 100). Thus, a 10% score increase indicated that the removal of said variable decreased catchment sensitivity or exposure by 10%. Removing sensitivity or exposure variables always resulted in a decline in sensitivity or exposure of HUC12s relative to the original score, which included all metrics. We also removed all HUC12s with no wells and repeated the analysis to determine the contribution of UOG to HUC12 exposure and vulnerability.

#### Sensitivity, exposure, and vulnerability analysis

HUC12s were used as replicates within each shale play to compare differences in sensitivity, exposure, and vulnerability among plays. Mean scores were compared with parametric Analysis of Variance followed by Tukey’s post-hoc pairwise comparisons after checking for distribution and variance. Due to multiple comparisons, statistical significance was inferred at α = 0.016 (α = 0.05/3 variables).

## Results

### Across all plays summary

Environmental conditions varied across HUC12s within plays and clear geographic trends in those conditions were apparent based on the mapped results of the exploratory cluster analysis (Fig 1). General characteristics attributed to each cluster group based on descriptive statistics (S2 Table) provided a visualization of predominant trends in sensitivity and exposure variables, including a cluster group of HUC12s where high well density was a characteristic feature (Cluster 8, Fig 1). Hilliard (WY), Mowry (WY), and Barnett (TX) were the most similar plays with larger and less sloped catchments, areas of extensive grassland and low annual average precipitation. Environmental conditions varied substantially within the other three plays and are described in a later section for each play.

When evaluated across all plays precipitation averaged 845 mm, stream density averaged 0.80 km km^-2^, mean catchment slope was 4.3%, soil erodibility was 0.29 and the percentage of unconsolidated sediments was 5.4% (Table 3). Catchments were on average covered mostly by forest/grassland (64.8%), followed by pasture (13.9%), cultivated crops (9.6%), wetlands (2.2%) and then impervious surface (1.6%). Both mine and dam density averaged 0.01 per km^2^ and road density averaged 2.3 km km^-2^. Vertical and non-vertical well density averaged 0.14 and 0.06 wells km^2^, respectively, and was on average 406.5 and 422.5 meters from the nearest stream (Table 3). When removed from the overall score, any one variable that defined catchment sensitivity reduced HUC12 sensitivity by at least 10%, except the removal of unconsolidated sediment that decreased sensitivity an average of 6% (Table 4). All of the over 5000 catchments ranked had roads and impervious surfaces (Table 3). Crop and pasture were present in over 80% of all catchments, while vertical wells were in 40% and non-vertical wells in 20% of all catchments (Table 3). Removal of wetlands and mean catchment slope affected the total sensitivity score the most (Table 4). The removal of road density and pasture affected average overall exposure scores the most where scores decreased by 19 and 17%, respectively (Table 4).

**Table 3:** Summary statistics for HUC12 natural sensitivity and anthropogenic stressor exposure variables across all plays and within each play.

	All (n = 5921)		Bakken (n = 1060)		Barnett (n = 731)		Fayetteville (n = 211)		Hilliard (n = 526)		Marcellus (n = 3175)		Mowry (n = 215)	
*Sensitivity variables*	Mean	% non-zero HUCS	Mean	% non-zero HUCS	Mean	% non-zero HUCS	Mean	% non-zero HUCS	Mean	% non-zero HUCS	Mean	% non-zero HUCS	Mean	% non-zero HUCS
Precipitation (mm)	845.36	100	404.17	100	809.30	100	1282.58	100	343.74	100	1089.78	100	337.59	100
% Forest ^1^	64.82	100	42.30	100	77.61	100	57.98	100	93.96	100	62.90	100	95.98	100
% Wetlands^2^	2.18	86	3.51	100	0.72	85	2.30	100	1.39	84	2.29	81	0.99	99
Stream density (km/km2)	0.80	99	0.90	100	0.65	100	1.07	100	0.71	87	0.79	100	0.84	100
Mean slope (degrees)	4.28	100	1.69	100	1.57	100	4.09	100	4.25	100	5.90	100	2.64	100
Soil erodibility (kfactor)	0.29	100	0.30	100	0.28	100	0.29	100	0.25	98	0.30	100	0.28	100
% Loose sediment	5.41	32	7.47	45	15.63	85	12.58	40	14.31	78	0.40	5	5.54	55
*Stressor variables*														
Well density (vertical, wells km^-2^)	0.14	40	0.02	22	0.06	37	0.15	45	0.09	47	0.19	45	0.33	54
Mean distance of vertical wells to streams (m)	406.52	40	797.49	22	472.09	37	322.32	45	491.63	47	325.42	45	370.95	54
														
Well density (non-vertical, wells km^-2^)	0.06	20	0.07	16	0.15	20	0.26	35	0.06	19	0.03	21	0.01	23
Mean distance of non vertical wells to streams (m)	422.50	20	718.60	16	458.31	20	258.27	35	492.34	19	352.98	21	335.53	23
Road density (km km^-2^)	2.30	100	1.24	100	2.26	100	0.85	100	0.61	100	3.07	100	1.83	100
Mine density (# km^-2^)	0.01	32	0.00	13	0.00	16	0.02	50	0.02	48	0.01	36	0.04	55
Dam density (# km^-2^)	0.01	47	0.01	32	0.02	69	0.01	54	0.00	21	0.01	49	0.01	59
% Cultivated crops	9.64	87	4.54	78	3.28	79	25.48	100	2.35	54	13.60	99	0.33	41
% Impervious surface^3^	1.61	100	0.40	100	2.60	100	1.21	100	0.34	100	2.11	100	0.27	100
% Pasture ^4^	13.90	86	42.98	99	6.09	95	5.52	77	0.12	9	9.74	96	0.54	34

^1^(NLCD class 41 + 42 + 43 + 52 + 71).

^2^(NLCD class 90 + 95).

^3^(NLCD class 81).

^4^(NLCD class 82).

**Table 4:** Average HUC12 percent change (%Δ, overall average sensitivity or exposure-sensitivity or exposure with variable removed *100) and the standard deviation (stdev) in change. Removing a variable from the overall scoring always reduced catchment sensitivity or exposure values (see Methods).

	All plays		Bakken		Barnett		Fayetteville		Hilliard		Marcellus		Mowry	
	%Δ	SD	%Δ	SD	%Δ	SD	%Δ	SD	%Δ	SD	%Δ	SD	%Δ	SD
*Sensitivity metric removed*														
Precipitation (mm)	14	6	20	3	16	2	6	1	19	3	11	4	20	3
% Forest/Grassland ^1^	16	5	12	3	17	5	15	5	19	4	17	5	20	3
% Wetlands^2^	18	10	21	6	13	7	20	6	12	7	18	11	15	4
Stream density (km/km^2^)	16	5	15	5	13	4	19	5	11	6	18	4	14	4
Mean slope (degrees)	17	6	11	2	11	1	17	5	14	4	21	6	11	2
Soil erodibility (kfactor)	16	5	16	5	13	3	17	5	11	3	18	5	13	4
Proportion of unconsolidated sediment	6	9	8	9	17	8	8	10	15	8	1	4	9	8
*Exposure variables*														
all UOG metrics	6	11	5	9	6	9	13	15	8	12	6	11	8	11
Well density (non-vertical, wells/km^2^)	4	8	3	8	4	7	7	10	5	10	3	7	5	9
Mean distance of non-vertical wells to flowlines (m)	2	5	2	5	2	5	6	9	3	7	2	5	4	8
Well density (vertical, wells/km^2^)	8	10	4	7	7	10	10	11	12	14	8	9	11	11
Mean distance of vertical wells to flowlines (m)	7	9	4	9	7	10	8	9	12	14	7	8	11	12
Road density (km/km^2^)	19	7	18	6	17	7	13	7	20	10	19	6	19	11
Mine density (#/km^2^)	7	11	3	8	3	8	10	10	14	16	7	10	13	12
Dam density (#/km)	10	11	7	11	16	12	10	10	5	10	10	11	13	12
% Cultivated crops	12	7	12	8	11	7	15	4	9	10	14	5	6	7
% Impervious surface^3^	15	6	16	5	16	6	13	4	19	10	14	5	15	7
% Pasture ^4^	17	11	30	10	17	9	10	8	1	4	16	7	5	7

^1^(NLCD class 41 + 42 + 43 + 52 + 71).

^2^(NLCD class 90 + 95).

^3^(NLCD class 81).

^4^(NLCD class 82).

**Fig 2:**
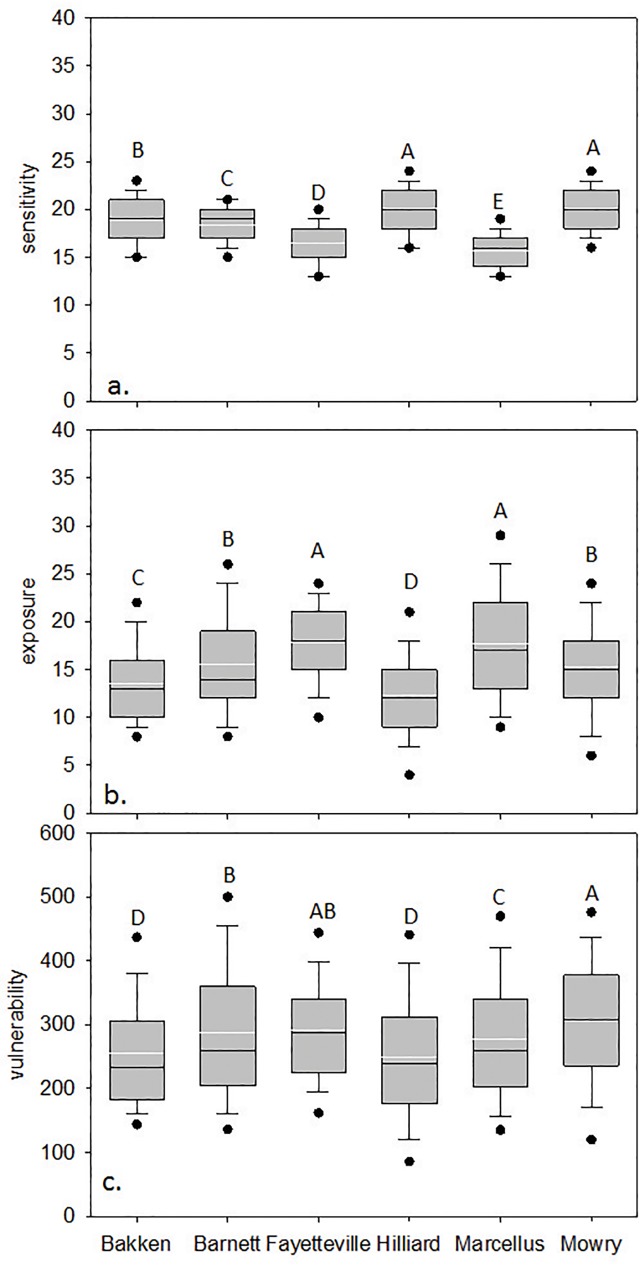
Average HUC12 scores in each shale play where a. is potential natural sensitivity of HUC12s to stressors, b. is existing HUC12 exposure to multiple stressors, and c. is the computed vulnerability (mean HUC12 sensitivity x exposure to multiple stressors). Dots are 95^th^ percentiles, whiskers are upper 25^th^ and lower 75^th^ percentiles, solid black line is median and white solid line is mean. Letters above box and whisker plots indicate significant differences (p ≤ 0.016).

**Fig 3:**
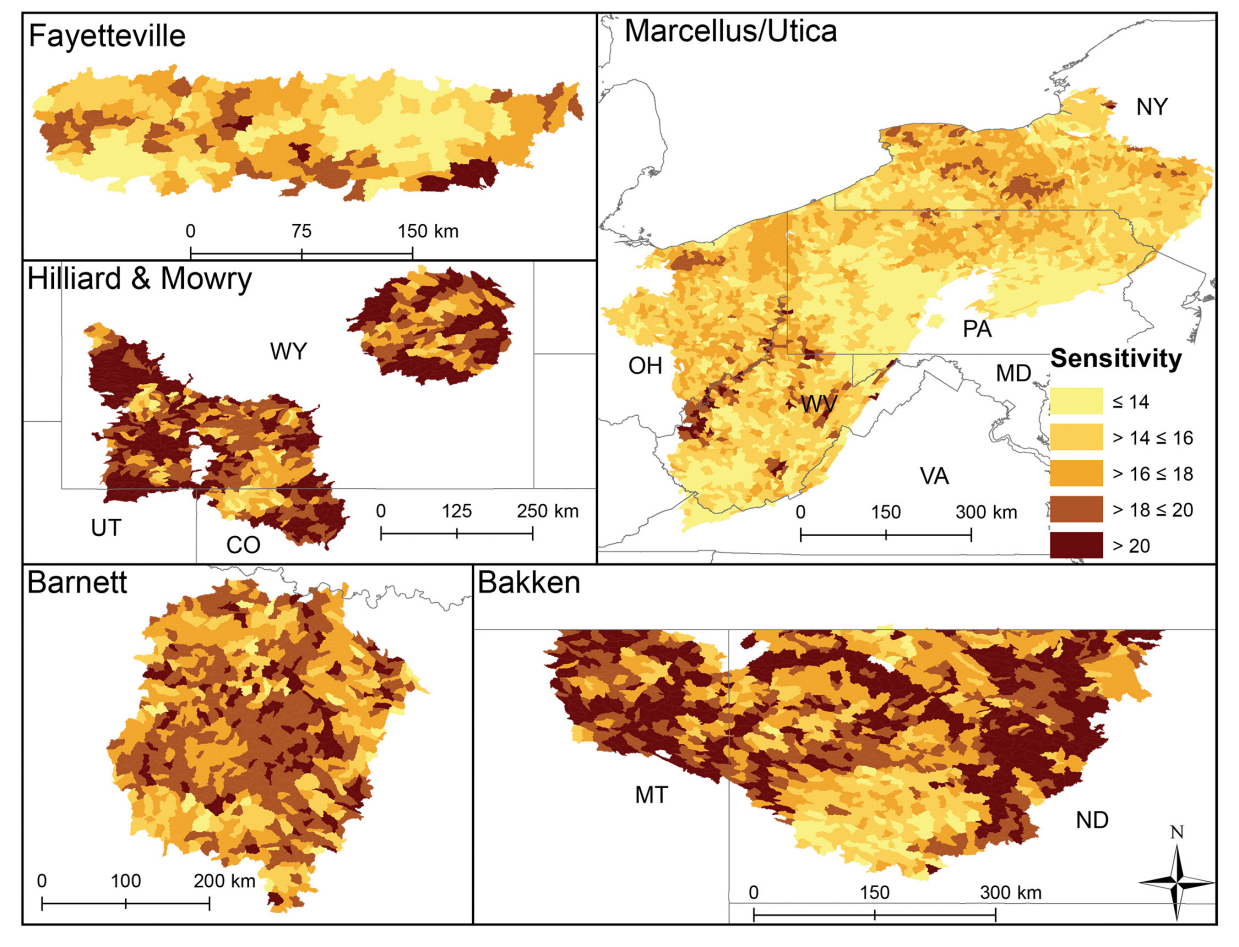
HUC12 sensitivity scores that represent natural characteristics in each shale play. Lighter colors illustrate catchments predicted to be less sensitive to stressor exposure.

At the shale play level catchment sensitivity significantly differed (p<0.0003, Fig 2A), even though the range in average catchment sensitivity was narrow (ranging from 15–20). Mowry and Hilliard HUC12s were on average the most sensitive, followed by Bakken, the Barnett, the Fayetteville and finally the Marcellus HUC12s (Figs 2A and 3). HUC12 average sensitivity tended to align with Level I ecoregions where catchments within the North American Desert and Great Plains were most sensitive and catchments in the Eastern Temperate Forests were least sensitive.

The individual variables driving catchment sensitivity differed among plays. For example, Bakken catchment sensitivity scores were driven by low precipitation and wetland cover and Marcellus catchment sensitivity was driven by catchment slope. Low annual average precipitation and high grassland cover explained sensitivity of catchments in Hilliard and Mowry HUC12s (Tables 3 and 4). In contrast, HUC12s in the Barnett were sensitive due to extensive unconsolidated sediment and high proportion of grasslands. While all catchments had extensive vegetation, deciduous forest covered less area within Marcellus and Fayetteville than grassland covered in Mowry and Hilliard plays (Table 3). Removal of any one sensitivity variable from the total sensitivity scored changed the score by 1 to 21%. These results are further discussed by play below.

**Fig 4:**
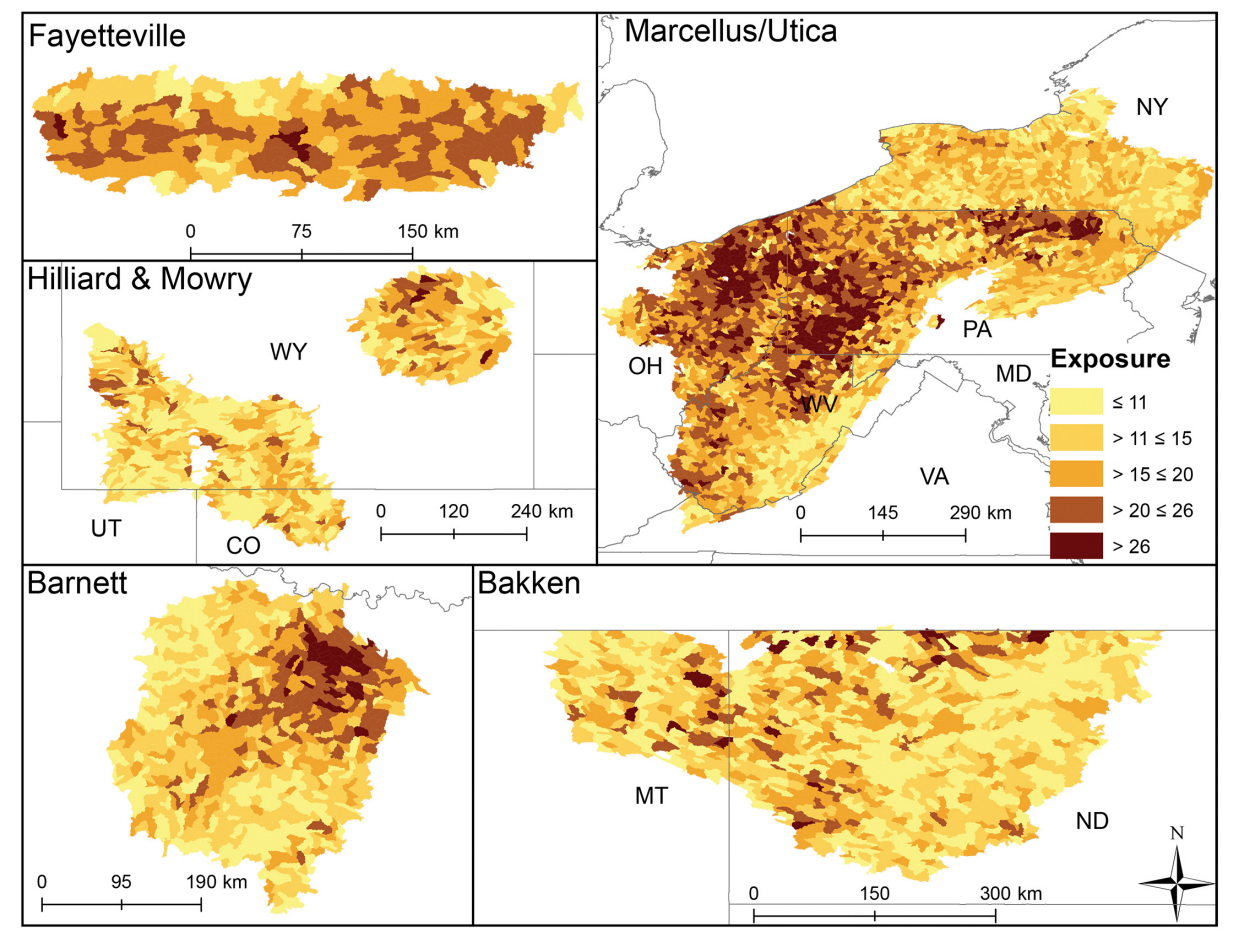
HUC12 exposure scores to multiple stressors in each shale play. Lighter colors illustrate catchments predicted to have experienced less stress from lower exposure to multiple stressors.

**Fig 5:**
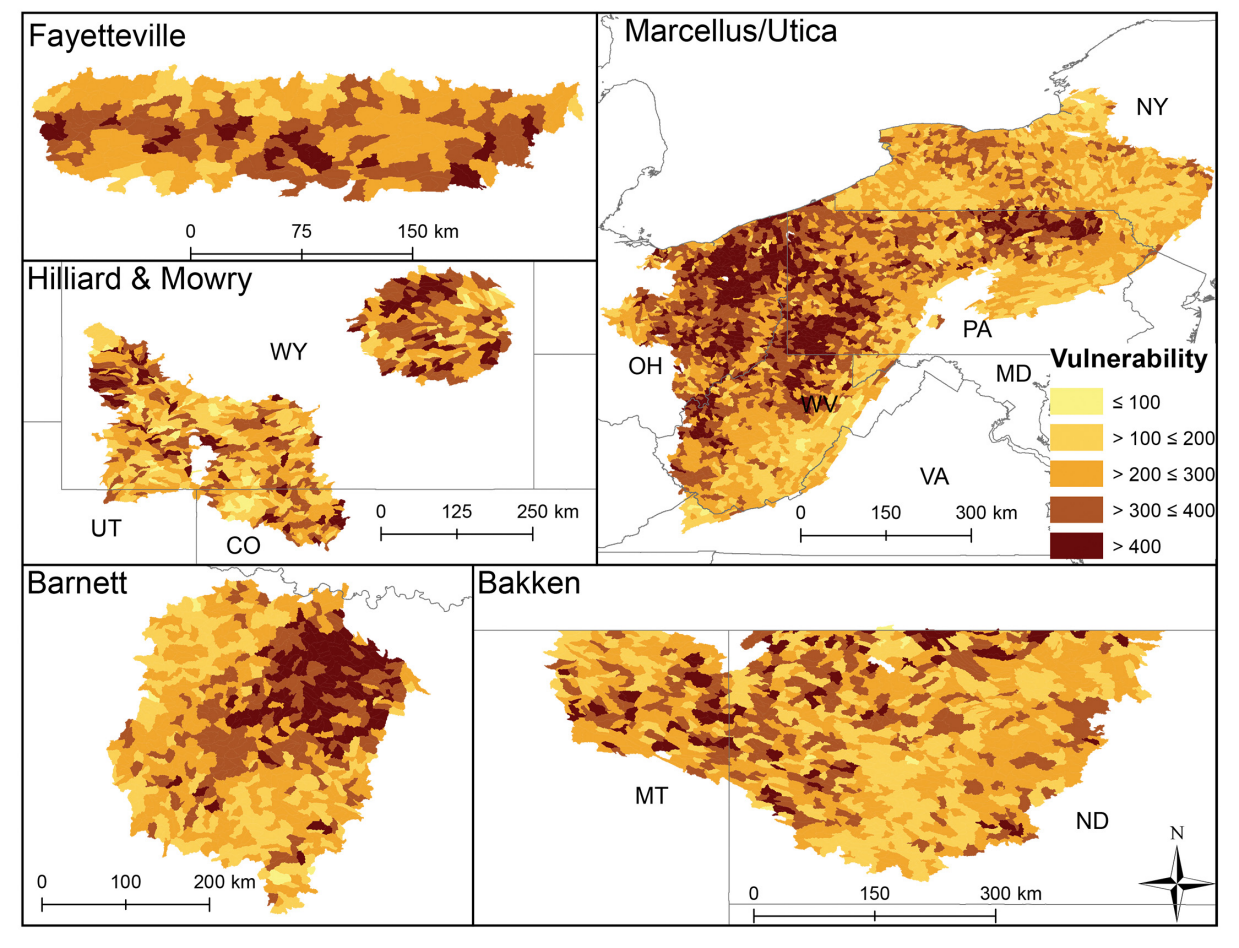
HUC12 vulnerability scores (sensitivity x exposure) in each shale play. Lighter colors illustrate lower values or lower vulnerability. Greater vulnerability was predicted to indicate greater potential for biological degradation with future development; however, some catchments may already have suffered significant species loss from multiple pre-existing stressors. Such loss may have resulted in a community dominated by tolerant species and thus be less vulnerable to future development than what is presented. Biological data are needed to resolve this issue.

Average exposure scores also significantly differed among plays (p < 0.003, Fig 2B) and varied more across and within plays (Figs 2 & 4) than sensitivity and did not align with Level I ecoregions. Eastern HUC12s (Marcellus, Fayetteville, and Barnett) generally had more cumulative stressors with greater intensity that resulted in higher exposure scores than other plays (Fig 2B). Average catchment road density was highest in the Marcellus play, average mine density was highest in the Mowry, and highest average catchment dam density and impervious surface cover were in the Barnett. The Fayetteville had the highest average cover of row crops and catchments in the Bakken had on average the highest cover of pasture (Table 3). Vertical well density was highest in the Mowry; mean distance of vertical and non-vertical well to streams and non-vertical well density was highest in the Fayetteville. The removal of any one exposure variable tended to have less impact on scores than it did for the sensitivity scores. Exposure scores declined by 5–13% on average across plays when well density and well proximity were removed (Table 4). Catchment vulnerability differed among the plays (p < 0.003, Fig 2C) with catchments in the Mowry being on average most vulnerable and catchments in the Bakken and Hilliard being least vulnerable. HUC12s within plays with greater sensitivity and exposure to stressors ranked as most vulnerable (Figs 2C and 5).

**Fig 6:**
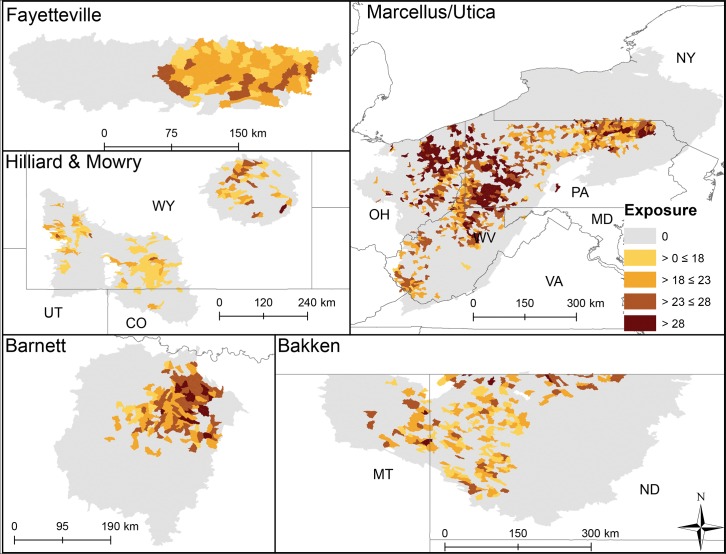
HUC12 exposure scores that include only UOG well density and proximity for each across shale plays.

**Fig 7:**
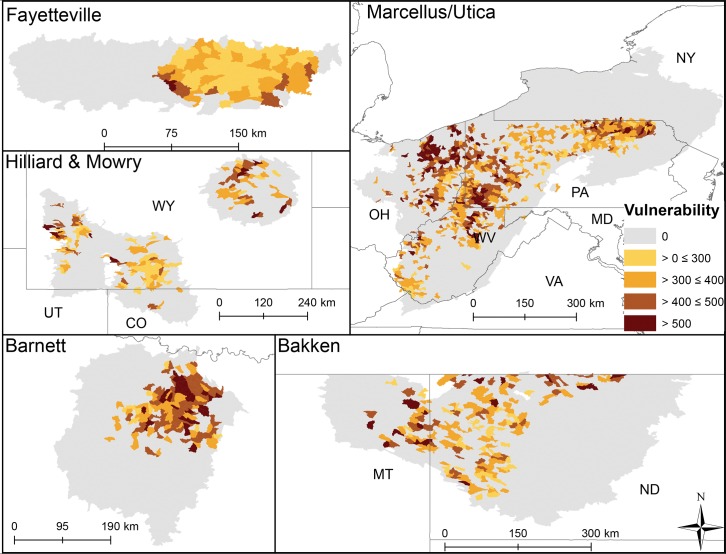
HUC12 vulnerability (natural sensitivity x stressor exposure) calculated from exposure scores that only include UOG well density and proximity across each shale play. Lighter colors illustrate lower values or lower vulnerability. Greater vulnerability was predicted to indicate greater potential for biological degradation with future development; however, some catchments may already have suffered significant species loss from pre-existing stressors. Such loss may have resulted in a community dominated by tolerant species and thus be less vulnerable to future development than what is presented. Biological data are needed to resolve this issue.

We also ranked exposure of all HUC12s by UOG activities by calculating total exposure only defined by UOG well density and well proximity to flowlines. UOG-only exposure scores were then multiplied by companion sensitivity scores to generate UOG-only vulnerability ranks. UOG-only exposure scores were consistently high due to density of UOG wells in catchments where activity was present and from differences in well proximity to flowlines (Fig 6). UOG-specific vulnerability varied more within plays due to differences in HUC12 sensitivity to disturbances (Fig 7). All HUC12 environmental values are provided in S3 Table.

### Bakken

Vertical well densities were lower in the Bakken than the across-play average (mean = 0.02 versus 0.14 wells km^-2^, respectively), but non-vertical (0.07 wells km^-2^) densities were greater than the across-play average (0.06 wells km^-2^). Despite the intensity of oil and gas development over the last 5 years, wells tended to be set farther back from NHD flowlines (mean 718.6 m for unconventional and 797.4 m for vertical) and spaced farther apart than in other plays. This region has a low annual mean precipitation (404 mm per year) and a relatively high density of streams (0.9 km km^-2^), wetlands (3.5%), and unconsolidated sediment (7.5%). Extensive and relatively high density of wetlands and unconsolidated soils resulted in the Bakken being one of the more sensitive plays (Fig 2A). However, average HUC12 exposure to anthropogenic stressors was lowest among plays evaluated (Fig 2B). Roads, crops, and impervious surfaces were present in all catchments, but less dense in these catchments compared to the across play average (Table 3). Pasture cover was the only exposure that was both extensive and intense (mean = 43%) relative to exposure scores in the other plays (Fig 1, Table 3). Medium sensitivity and low exposure resulted in the Bakken being one of the least vulnerable plays (Fig 2C). HUC12 sensitivity, exposure and vulnerability did not show clear geographic trends (Figs 3–5). The removal of pasture from our exposure scores had the greatest effect (30%), while removing wetlands (21%) and average 30 year precipitation (20%) affected sensitivity scores the most. By removing UOG well density and proximity from the analysis, exposure values declined by 5% because although wells were dense, they were concentrated in a relatively small area of the play (Fig 6, Table 4). UOG specific exposure and vulnerability were variable in this play (Figs 6 &7). Of all six plays, the Bakken was most vulnerable to alterations in the natural flow regime and 3^rd^ most vulnerable to sedimentation and chemical stressors (Table 5).

**Table 5:** Average HUC12 severity-weighted vulnerability (natural sensitivity x stressor exposure) in each play to most common potential stressors associated with UOG from Souther et al. 2014. Plays were ranked from 1(low) to 6 (high) based on their HUC12 means relative to the across-play means from Table 3 and then selected exposure variables were multiplied by author-derived severity scores that ranged from 1 (least severe) to 3 (most severe). Summed sensitivity ranks were multiplied by severity-weighted exposure scores to derive a total. See Paukert et al. 2011 for a more detailed discussion of this method. Severity scores are in parentheses and sensitivity ranks are bold.

Natural flow regime	Bakken	Barnett	Fayetteville	Hilliard	Marcellus	Mowry
**Precipitation (mm)**	**4**	**3**	**1**	**5**	**2**	**6**
**Stream density (km/km** ^**2**^ **)**	**5**	**2**	**6**	**1**	**3**	**4**
Well density (vertical, wells/km2) (1.25)	1	3	5	4	6	8
Well density (non-vertical, wells/km^2^) (2.00)	8	10	12	6	4	2
Road density (km/km^2^) (1.33)	4	7	3	1	8	5
Mine density (#/km^2^) (1.80)	4	4	7	7	5	11
Dam density (#/km) (3.00)	6	18	6	3	6	6
% Cultivated crops (2.50)	10	8	15	5	13	3
% Impervious surface (3.00)	9	18	12	6	15	3
% Pasture (1.42)	9	6	4	1	7	3
Total	459	370	448	192	320	410
Sediment						
**Mean slope (degrees)**	**6**	**4**	**3**	**2**	**5**	**1**
**% Unconsolidated sediment**	**3**	**6**	**4**	**5**	**1**	**2**
**Soil erodibility (k factor)**	**6**	**3**	**4**	**2**	**6**	**3**
Vertical well proximity to flowlines	1	3	6	2	5	4
Non-vertical well proximity to flowlines	4	5	6	3	2	1
Well density (vertical, wells/km^2^) (1.25)	2	4	9	7	11	13
Well density (non-vertical, wells/km^2^) (2.50)	9	11	13	7	4	2
Roads (2.75)	8	14	6	3	17	11
Mine density (#/km^2^) (1.80)	4	4	7	7	5	11
Dam density (#/km) (1.70)	3	10	3	2	3	3
% Cultivated crops (2.67)	11	8	16	5	13	3
% Impervious surface (2.17)	7	13	9	4	11	2
% Pasture (1.83)	11	7	5	2	9	4
Total	889	1028	880	371	970	323
Chemical						
**Mean slope (degrees)**	**6**	**4**	**3**	**2**	**5**	**1**
**% Unconsolidated sediment**	**3**	**6**	**4**	**5**	**1**	**2**
Vertical well proximity to flowlines	1	3	6	2	5	4
Non-vertical well proximity to flowlines	4	5	6	3	2	1
Well density (vertical, wells/km^2^) (1.67)	2	3	7	5	8	10
Well density (non-vertical, wells/km^2^) (2.33)	9	12	14	7	5	2
Roads (2.33)	4	7	3	1	8	5
Mine density (#/km^2^) (3.00)	4	4	7	7	5	11
Dam density (#/km) (1.00)	6	18	6	3	6	6
% Cultivated crops (2.20)	10	8	15	5	13	3
% Impervious surface (2.83)	9	18	12	6	15	3
% Pasture (1.70)	9	6	4	1	7	3
Total	514	824	558	287	444	143

### Barnett

Non-vertical well density was second highest in this play (0.15 wells km^-2^) with most development occurring in the densely populated Dallas/Fort Worth urban area (Figs 1 and 6). In 2009, 1169 non-vertical and ~2400 vertical wells were drilled compared to 3153 and 944 in 2011. Barnett had slightly lower average annual precipitation (809 mm) than the across-play average. This together with the Barnett having above study-average forest/grassland (77.6%) and unconsolidated sediment (15.6%) resulted in the Barnett ranking 4^th^ in sensitivity of all plays (Fig 2A). A relatively high density of non-vertical wells coupled with greater than average impervious surface (2.6%) and dam density (0.02 #/km) resulted in it having a relatively large exposure score–tied with Mowry and behind the Fayetteville and Marcellus catchments (Fig 2B). Little spatial pattern was evident in sensitivity scores (Fig 3), whereas greater exposure and vulnerability scores were concentrated in the Northeast (Dallas/Fort Worth area, Figs 4 and 5). When we removed variables from sensitivity scores, unconsolidated sediment (17%) and forest/grassland (17%) reduced sensitivity scores the most. Removing road density and pasture reduced average HUC12 exposure scores the most (both 17%, Table 4). UOG-specific exposure and vulnerability were consistently high across catchments in this play (Figs 6 and 7) and when these specific exposures were removed, exposure scores declined on average by 6% (Table 4). Of all six plays, the Barnett was most vulnerable to sedimentation and chemical stressors and 4^th^ most vulnerable to altered flow regime (Table 5).

### Fayetteville

Average non-vertical well density (0.26 wells km^-2^) was greatest in the Fayetteville shale (Table 3). The Fayetteville play also had the highest annual mean precipitation (mean = 1282 mm) and row crop (25.5%, Table 3). Sensitivity of catchments in this play ranked overall as 5^th^ most sensitive (Fig 2A). Sensitivity was driven by a greater than average wetland coverage (2.3%), stream density (1.07 km km^-2^) and unconsolidated sediment (12.6%). In contrast, the exposure scores tended to be greater in these catchments than in other plays tying it for the most exposed with the Marcellus (Fig 2B). HUC12 average mine density (0.02 # km^-2^) and vertical well density (0.15 km km^-2^) tended to be greater than in other plays and mean vertical and non-vertical distance of wells to streams (322.3 and 258.2 m, respectively) were closer than in other plays (Table 3). Combined sensitivity and exposure scores resulted in the 2^nd^ highest average vulnerability score among the plays (Fig 2C). Spatial patterns showed a low sensitivity for HUC12s in the northeast part of the play (Fig 3), whereas exposure and vulnerability showed no clear pattern (Figs 4 and 5). Removing wetlands or stream density from our analysis resulted in average decreased sensitivity by 20 and 19%, respectively. Crop cover had the largest effect on average exposure scores (15%, Table 4). The combined removal of non-vertical well density and proximity scores affected average exposure scores the most in this play reducing exposure by an average of 13% when removed. UOG-related exposure was relatively high and vulnerability was relatively low compared to HUC12s in the Barnett and Marcellus (Table 4, Figs 6 and 7). Of all six plays, the Fayetteville was 2^nd^ most vulnerable to alterations in the natural flow regime, 4^th^ most vulnerable to sedimentation and 2^nd^ most vulnerable to chemical stressors (Table 5).

### Hilliard-Baxter-Mancos

Non-vertical well density reflected the average across plays (0.06 wells km^-2^), but it had an extremely high co-efficient of variation (709%) with UOG development currently concentrated in south-central and south-west Wyoming (Figs 1 and 6, Table 3). Catchments in the Hilliard play scored as most sensitive along with Mowry (Fig 2A) due to little rainfall (344mm), extensive forest/grassland (94.0%), and unconsolidated sediment (14.3%, Table 3). The Hilliard ranked as the least exposed to cumulative stressors (Fig 2B). Impervious surfaces and roads occurred in all HUC12s, but Hilliard average values were near the lowest among the plays (Table 3). The only exposure that was at or above the whole study average was mine density (0.02 #/km^-2^). Despite its high sensitivity the Hilliard play ranked as one of the least vulnerable plays (Fig 2C). This low vulnerability ranking reflects, in part, that while the Hilliard play has the highest total recoverable estimates of shale gas after the Appalachian basin, it has not yet been extensively developed (Table 1, Fig 6). Relatively high sensitivity scores occurred throughout this play (Fig 3) whereas exposure scores were mostly low (Fig 4). Overall HUC12 vulnerability showed no clear spatial patterns (Fig 5). Variables with the greatest effect on scores were impervious surfaces or road density for exposure (19 and 20% change in average exposure) and forest/grassland cover or precipitation for sensitivity (both 19%; Table 4). Removal of UOG-exposure reduced catchment exposure by 8% where it tended to be relatively low with HUC12 in the northwest and vulnerable from catchment sensitivity (Table 4, Figs 6 and 7). Of all six plays, the Hilliard was the least vulnerable to alterations in the natural flow regime and sedimentation and 2^nd^ least vulnerable to chemical stressors (Table 5).

### Marcellus-Utica-Devonian

Overall non-vertical density was below the across-play mean (0.03 wells km^-2^) (Table 3) because large areas underlain by Marcellus have not been developed due to legal restrictions (New York State) or to limited gas-development potential. Non-vertical wells were on average closer to NHD flowlines than the across-play average (Table 3). Much of the Marcellus underlies the Appalachian basin with extensive deciduous forest and catchments in this play had on average 62.9% forest cover. The remainder was predominantly row crops and pasture. Despite relatively widespread forest cover, catchments in the Marcellus play were ranked less sensitive to disturbance than catchments in all other plays (Fig 2A). Sensitivity characteristics were highest among all HUC12s for sloped catchments (5.9%), and erosive soils (0.3), and above the across-play average for wetland cover (2.29%, Table 3). In contrast, catchments in the Marcellus had the lowest proportion of unconsolidated sediments (0.4%) and a relatively high annual average precipitation (1089.7mm). The Marcellus tied with the Fayetteville as being the most exposed (Fig 2B) due to a high road density (3.07 km km^-2^), impervious surface (2.1%), crop cover (13.6%), and vertical well density (0.19 wells/km^2^) together with a relatively close proximity of vertical and non-vertical proximity to NHD flowlines (325.4 m and 352.9 m, respectively). Marcellus HUC12s ranked, on average, as 4^th^ most vulnerable from high exposure scores and low sensitivity (Fig 2C). Spatial patterns in sensitivity for this play showed some highly sensitive HUC12s in West Virginia and moderately sensitive catchments in New York (Fig 3). HUC12s with high exposure and vulnerability scores centered in northeastern and western Pennsylvania and throughout the Ohio, Maryland and northern West Virginia portions (Figs 4 and 5). Removal of average catchment slope resulted in the greatest change in sensitivity (21%). Removing road density changed exposure scores the most (19%; Table 4). Removal of non-vertical well density and proximity to NHD flowlines changed the exposure score by 6%. Marcellus currently has the most catchments with UOG activity and many catchments with high UOG-related vulnerability (Figs 6 and 7). Of all six plays, the Marcellus was 5^th^ most vulnerable to alterations in the natural flow regime, 2^nd^ most vulnerable to sedimentation (tied with Fayetteville) and 4^th^ most vulnerable to chemical stressors (Table 5).

### Mowry

The Mowry play was unusual in that it had the highest average density of vertical wells (0.33 wells km^-2^), but the lowest average density of non-vertical wells (0.01 wells km^-2^). Over 90% of vertical wells were producing coalbed methane at the end of 2013. Catchments in this play tied those in the Hilliard as most sensitive due to being semi-arid (30 yr mean precipitation = 338mm) with extensive forest/grassland (96.0%, Fig 2A) and having a relatively high stream density (0.84 km km^-2^) and unconsolidated sediment (5.5%; Table 3). Exposure scores were surprisingly high, ranking 3^rd^ along with Barnett (Fig 2B), mostly from high vertical well density, close proximity of both vertical (370.9m) and non-vertical wells to NHD flowlines (335.5m), and a high density of mines (0.04 #/km^-2^; Table 3). No clear spatial patterns in HUC12 sensitivity, exposure or vulnerability were evident (Figs 3, 4 and 5). Sensitivity scores were most affected by precipitation (20%) and % grassland/forest (20%), while exposure scores were most affected by road density and % impervious surface (19 and 15%, Table 4). Removal of non-vertical well density and proximity to NHD flowlines changed the exposure score by 8%. UOG well density was relatively low reflected by lower ranked UOG-exposure (Fig 6); however, natural catchment sensitivity resulted in greater vulnerability than many other plays (Fig 7). Of all six plays, the Mowry was 3rd most vulnerable to alterations in the natural flow regime and chemical stressors and 2^nd^ to least vulnerable to sedimentation (Table 5).

## Discussion

### Patterns in catchment sensitivity, exposure, and vulnerability across plays

Within-play vulnerability scores should be more useful for resource managers that operate on local scales, while the across-play analysis provides added context for discussing potential ecological impacts across geographic boundaries that can guide future research. Our catchment vulnerability analysis highlighted stream catchments where water quality and biological communities are more likely to be degraded from natural characteristics interacting with current anthropogenic activities. Catchment sensitivity, exposure, and vulnerability varied within and across plays (Figs 3–5). Overall, forest/grassland and wetland most commonly contributed to catchment sensitivity across the plays; however, across-play differences were instructive. For example, wetlands contributed most to catchment sensitivity in Bakken catchments while slope contributed most in the Marcellus. Road density was the anthropogenic exposure that affected across-play average catchment exposure scores the most in five of the six plays, although pasture and row crop agriculture were also important exposures. UOG development was not a spatially extensive stressor (20% of all HUC12s); however, when it was present it tended to be in high density and resulted in greater catchment vulnerability.

Catchment sensitivity and exposure scores and their resultant composite vulnerability score can be used to highlight catchments where the effects of UOG development (or other existing exposures or currently unanticipated pressures) are most probable, guide best management practices, highlight data gaps, and prioritize monitoring efforts. The maps from our analyses (Figs 2–7) can be used to guide such an analysis. For example, catchment sensitivity can be used to predict streams most vulnerable to future infrastructure development, water sourcing for drilling and UOG development, potential for water use allocations to change, or where longer recovery time from accidental contaminant spills or leaks might occur. Alternatively, regions with extensive infrastructure can be identified where fewer impacts may occur. For example, catchments in the Barnett play have extensive roads that will limit the need for additional development compared to the Bakken where news roads are more likely to be needed. Our analysis could also be applied to stream reach-scales for site-specific research and resource management. By taking environmental context into account as the landscape is being modified, it should be possible to predict whether lessons learned in one play could be transferable to another play.

Our vulnerability metric does not include future development potential, but it can be used to anticipate vulnerability. The Mowry play ranked as highly vulnerable, but it does not have high near-future development potential and its energy development is dominated by coalbed methane for which production has slowed considerably. However, the knowledge that natural catchment features rather than existing exposures make catchments in this play vulnerable could be used to guide development of any kind in the future. In contrast, catchments in the Marcellus ranked as vulnerable from a combination of catchment sensitivity characteristics and existing exposures. Therefore, anticipated build-out from UOG will interact with other exposures, which could have unanticipated environmental effects. Marcellus, Hilliard, and Bakken plays are projected to have the greatest build-out in the next decade and our analysis can be used to target catchments most likely to be vulnerable in the future for analysis before activity begins as a way to identify possible cumulative ecological effects.

### Stream catchment vulnerability to water stress, sedimentation, and chemical contamination

Streams that drain sensitive catchments are expected to experience more rapid degradation from UOG or other anthropogenic development [36]. Entire shale plays and catchments within plays could be more prone to ecological degradation from specific activities associated with UOG. Water stress, potential for chemical contamination into a river system, on- and off-site spills and leaks of waste fluids, siltation, and cumulative effects from all of these exposures vary with natural conditions of a catchment (Table 5) [31,45]. For example, Table 5 indicates play-specific stream vulnerability to water stress or sedimentation from activities associated with UOG will be exacerbated by existing agriculture and impervious surfaces. For example, because dams increase water residence time, their presence could interact with chemical contamination to amplify stream ecosystem effects in regions such as the Barnett.

Water consumption from UOG development is a widespread concern, especially in areas with low precipitation, or high demand from agriculture and municipal water use [46,47]. Based upon our analysis, catchments in the Bakken, Mowry, and Hilliard plays are more vulnerable to water stress from low annual average precipitation and catchments in the Marcellus and Fayetteville will be vulnerable from greater municipal and agricultural demand. Reduced water availability is also a concern when water is sourced from headwaters or intercepted by excavated holding ponds that can alter natural streamflow regimes and drinking water reservoirs [48]. A recent US EPA study in Pennsylvania where water stress should be low and Colorado where it should be high showed no water imbalance from UOG water sourcing of local surface waters [49]. In Texas, 0.2% of total Texas water supply was used for hydraulic fracturing in an already water stressed region in 2011 [50]. Furthermore, HUC12s in the Barnett have a high density of dams and impervious surfaces that already alter flow regimes. Water-use conflict in Western states will continue with UOG development and intensify with the changing climate. However, water sourcing from streams in regions with higher than average precipitation does not preclude water stress. For example, despite having the greatest annual precipitation, most rain falls in Arkansas late autumn and winter with little falling in late spring and summer. Consequently, a majority of streams (1^st^-5^th^ order) in Arkansas do not flow year round. Water extraction during low- and no-flow periods could alter habitat for resident invertebrates and fishes adapted to these intermittent stream conditions by reducing pool size and connectedness within the river network [51]. In addition, reduced streamflow can create physicochemical conditions (e.g., water temperature, oxygen levels) stressful to aquatic communities, such as native spawning fishes and aquatic invertebrates [39,52].

Catchment geology and vegetation coupled with more impervious surfaces or bare ground will mediate contaminant transfer to nearby streams [31]. Catchments in the Barnett and Hilliard have more unconsolidated sediments and impervious surface that can increase the amount and rate of vertical and horizontal movement of spilled or leaked contaminants to groundwater. Fayetteville catchments have a higher average density of streams and wells are closer to the stream channels. Streams closer to well pads may be more likely to experience contamination from accidental exposure [20]. Greater frequency and intensity of precipitation in the Marcellus and Fayetteville plays could also increase the probability of contaminants flushing into nearby streams or overflowing holding ponds. Intact ground cover and pad and reservoir linings should provide some mitigation of contaminant movement and reduce infiltration and the probability of contact with ground or surface waters. If contaminants do reach ground or surface waters, subsurface movement is difficult to predict and remediate. Thus contaminants are more likely to persist long-term in the environment. Furthermore, unknown contaminants moving through complex stream-groundwater interactions are likely not going to be detected or mitigated [7]. UOG development over shallow alluvial aquifers, as in the Barnett, Bakken, Mowry, and parts of the Marcellus plays could increase surface water risk of chemical contamination[53]. Based upon our vulnerability analysis, well pads have not been sited closer to stream channels to access water; therefore, catchments with greater stream channel density tend to have closer well pads, access roads, and pipelines except in the Bakken.

Release of inadequately treated waste water can elevate chloride and other conservative elements and has been a leading cause of surface water contamination from hydraulic fracturing [18]. Treatment of hydraulic fracturing fluids is declining in Marcellus States; however, recycled produced and flow back waters are being treated and discharged back into ephemeral semi-arid streams in the Mowry play, which changes the natural flow regime and potentially adversely affects biota [54]. The extent to which specific exposures contribute the most to increase surface water total dissolved solids and associated ions is currently unknown. However, there are several mechanisms by which UOG could contribute to elevated surface water dissolved solids. Soil disturbance from infrastructure development [55], accidental surface spills and leaks, and fluid migration from oils and gas wells or deep-well injection sites could all contribute to elevated dissolved solids [27,56]. Surface waters in plays where deep well injection is the most common waste water disposal method may have less potential for contamination; however, this risk analysis is incomplete [57]. Lethal effects of elevated chloride from road salts is well studied [58]. Elevated stream conductivity from formation water is a common ecological effect of resource extraction, and yet sub-lethal effects on biota are understudied [6,59]. Recent studies show that common mayfly taxa express slower growth and incomplete lifecycles at salt and calcium chloride concentrations found in surface waters following brine release [60,61]. Further studies are needed to understand possible ecosystem consequence of rising surface water salinity [62].

Sedimentation to surface waters, resulting from UOG development, can occur as a result of construction of well pads, access roads and pipelines [17]. Sedimentation is more likely when development is on a steep slope, close to a stream channel, soils are erosive, and other activities, such as roads and other impervious surfaces are present to transport materials. Our analysis shows that catchments in the Barnett, Bakken, Marcellus, and Fayetteville are more likely to experience sedimentation from land altering activities. Whether excessive sedimentation does occur will depend on the implementation and effectiveness of a suite of best management practices [63]. Sedimentation continues to be a leading source of surface water degradation despite known practices to reduce erosion, suggesting that effective best management practices tailored for different environmental conditions are needed [1].

### Implications for aquatic biota

The implications of energy development for aquatic organisms will depend both on the environmental context and the species present in vulnerable ecosystems. Our analysis provides insight into the environmental context component, for example, pointing to areas that might be vulnerable to surface disturbance and sedimentation. Our analysis has implications for species such as gravel spawning fishes and most aquatic insects that are highly sensitive to fine sediment accumulation [51,64]. However, our analysis does not take into account the number of species present and their sensitivity, which will vary among stream catchments and across shale plays [7]. Aquatic assemblage structure and diversity differ widely across the contiguous U.S. with the southeastern U.S. having the greatest fish, amphibians, crayfish, and mussel diversity where vulnerable streams in this region could suffer greater total species loss compared to ecosystems in the semi-arid Western states.[65]. The interpretation of which species are most sensitive and the potential for ecological ramifications might not always be straight-forward. While streams in semi-arid plays may have organisms more sensitive to lack of water, they may also have species that are adapted to intermittent drying. Likewise, many streams in the arid west have naturally high total dissolved solids and metals that biota are adapted to and therefore may be less vulnerable to these changes in water quality. In addition, the loss of one species could have more profound ecological impacts in species-poor ecosystems[66]. Our vulnerability analysis could be used to guide traditional bioassessment efforts that will refine and identify catchments with greatest vulnerability from both existing exposures such as urbanization and cultivated crops and the emerging exposures like UOG development.

### Limitations and utility of our analysis

Catchment scale analysis is reasonable when surface water impacts are of interest. However, the activities that make up UOG are myriad and may or may not adequately reflect potential for impacts. Currently, the density of active gas wells is the most accessible and defensible metric as a proxy for activity. This assumes that well pads are distributed evenly across the catchment and roads and pipelines correlate with pad density. However, well pads are typically square or rectangular disturbances that can be intentionally built at a distance from surface waters. State regulations typically prohibit construction of encroachments such as well pads into streams and wetlands without a permit, although many states provide exemptions if written justification are provided (e.g. Pennsylvania). In contrast, pipelines–and to a lesser extent, access roads are linear features. Pipeline construction typically involves stream crossings that introduce the likelihood of impacts such as sedimentation. Moreover, pipelines–especially midstream lines–can be located in catchments that have no well pads. While a few regional studies have shown that greater well density in a catchment correlated with more unpaved roads and greater density of pipelines [28,67], we cannot assume this is always the case. The likelihood of altered water quality from gas activities will also be determined to some extent by the vegetation and soil type interacting with the distance of an impact from the stream channel.

An additional limitation to our analysis is land use variables are incorporated in both the sensitivity (% wetlands, % forest/grassland) and exposure (% developed, % pasture, % crops) analyses and scores based on percentiles rather than known ecological effects. A catchment with a high percentage of developed/pasture/crop land will necessarily also have a low percentage of wetlands/forest/grassland resulting in a lack of complete independence of these analyses. In our results there were some plays where this trade-off was potentially occurring (e.g., Marcellus and Fayetteville), but there were other plays that were intermediate in both sensitivity and exposure (e.g., Bakken and Barnett) suggesting this did not have a large effect. Another potential concern with the land use data is that % forest + grassland was considered to be representative of natural or relatively undisturbed land. However, in the Western states much of what is categorized as grassland is likely heavily grazed and so actually experiences considerable disturbance. As a result the sensitivity of some Western plays may be slightly over-estimated. Although mostly vegetated, grassland and forest may not be natural from a legacy of past land use. In addition, there are regions that are less vegetated but could nonetheless be sensitive. We suggest State or local land cover data be used to compliment National Land Cover as appropriate. Furthermore, HUC12s are not independent units although our analysis treated each one as such. We recognize this flaw and suggest that the potential relationship among catchments be fully considered.

Our study also used the number of mines per catchment as an indicator of mining activity. As noted, mining has profound impacts on stream morphology and water quality due to practices such as mountaintop mining / valley fills and the discharge of abandoned mine drainage into surface waters [2,4,6]. A synthetic metric that focuses on acreage of mines, number and severity of acid mine drainage discharges, and length of stream channel impacted by mining in a catchment might give a better indicator of the impact of that exposure. Finally, our sensitivity and exposure scores were derived from a set of variables that were available at our scale of study; results must be interpolated with this caveat in mind. More localized variables may improve our estimates but were beyond the scope of this study.

### Conclusion

There is a need to define UOG trends in development across basins to identify surface waters most likely to have already experienced altered quality or those most at risk for degradation with future UOG development and other emerging exposures. Most studies currently focus on patterns and risk of effects that can be influenced by environmental context [57]. Our study provides a framework to generate testable hypotheses about where one might expect to see the greatest negative effects due to development-related surface disturbance or water use that could then be tested across a gradient of environmental contexts. For example, the rate at which sensitive species decline across a gradient of anthropogenic alterations in naturally sensitive catchments compared to less sensitive catchments will support catchment protection and best management practices. State and local agencies can select relevant spatially explicit data layers housed on a local website where users can define their own exposures and sensitivity variables to generate reach or catchment-scale areas of vulnerability. All shale plays, regardless of location, had a suite of catchments that spanned highly degraded to those that are less altered and naturally sensitive to alteration. Resource managers can use these catchments to identify monitoring and priorities for future development that reduces environmental effects by informing more effective best management practices.

## Supporting Information

S1 Table State gas well records were accessed from January 2000 to January 2013. Latitude, longitude, direction of bore (horizontal, vertical, directional), well type or class (gas, oil, etc), spud date and status (active or not) were included in the dataset and used to summarize well points.
(XLSX)

S2 Table An exploratory cluster analysis was performed to better visualize trends in “similar” environmental conditions across plays (i.e., combinations of the exposure/sensitivity variables per HUC12 detailed in Table 2). Descriptive statistics show unique characteristics of the ten cluster groups.
(XLSX)

S3 Table Environmental variables representing all HUC12s used in sensitivity and exposure analyses. (XLSX)
